# Adipofascial Infragluteal Perforator Flap for Total Parotidectomy Reconstruction: A Novel Application for Inconspicuous Donor and Recipient Site—Preliminary Results

**DOI:** 10.3390/jcm15072770

**Published:** 2026-04-06

**Authors:** Horațiu Rotar, Daniel Ostaș, Teodora Harina Iuga, Seong Gon Kim, Dragoș Țermure, Sergiu Samuilă, Lucian Fodor

**Affiliations:** 1 Department of Oral and Maxillofacial Surgery and Implantology, Faculty of Dental Medicine, “Iuliu Hațieganu” University of Medicine and Pharmacy, 400012 Cluj-Napoca, Romania; dragos.tarmure@gmail.com; 2 Department of Oral and Maxillofacial Surgery, Medicover Hospital Cluj-Napoca, 407062 Cluj-Napoca, Romania; daniel.ostas@gmail.com (D.O.); iugaharina@gmail.com (T.H.I.); 3 Department of Oral and Maxillofacial Surgery, College of Dentistry, Kangwon National University, Gangneung-si 25457, Republic of Korea; kimsg@gwnu.ac.kr; 4 Department of Plastic and Reconstructive Surgery, Bihor Emergency Clinical County Hospital, 410169 Oradea, Romania; sergiusamuila2@gmail.com; 5 Department of Plastic and Reconstructive Surgery, Interservisan, 400431 Cluj-Napoca, Romania; lucifodor@outlook.com

**Keywords:** adipofascial infragluteal perforator flap, total parotidectomy reconstruction, head and neck reconstruction

## Abstract

**Background:** Defects following total parotidectomy represent a distinctive reconstructive challenge. Restoration of facial volume and contour must be balanced with protection of the preserved facial nerve and reliable healing, particularly after extensive dissection and when adjuvant radiotherapy is anticipated. Multiple reconstructive options exist, each involving trade-offs regarding volume, pliability, long-term stability, and donor-site morbidity. We report our early clinical experience using the adipofascial infragluteal perforator (AIGP) free flap for reconstruction after total parotidectomy with skin and facial nerve preservation. **Methods:** We retrospectively reviewed the results of three consecutive patients undergoing total parotidectomy for parotid tumors, receiving immediate reconstruction with an AIGP free flap, operated between June and July 2025. The flap, based on terminal branches of the infragluteal vessels, was anastomosed to cervical recipient vessels. To address the two-compartment defect created by facial nerve preservation, the adipofascial tissue was tailored in a chimeric configuration to separately restore the superficial and deep parotid spaces. **Results:** All flaps survived. One patient developed a postoperative hematoma managed conservatively. Two patients developed minor donor-site seromas after drain removal, which resolved without intervention. Facial contour was satisfactorily restored in all cases, with mild overcorrection in one patient. Facial nerve function improved during follow-up. Donor-site scars were concealed within the infragluteal crease. **Conclusions:** In this preliminary case series, the AIGP free flap proved to be a feasible option for reconstruction after total parotidectomy with skin and facial nerve preservation, offering satisfactory contour restoration and low donor-site morbidity. Larger studies with longer follow-up are required to define indications and long-term outcomes.

## 1. Introduction

The parotid gland has a distinctive anatomy and tissue consistency, comprising superficial and deep lobes separated by the facial nerve. Reconstruction following oncologic parotid resection, particularly when the facial nerve is preserved, therefore presents unique challenges. Total parotidectomy results in two surgical dead spaces divided by a dissected and partially devascularized facial nerve and produces a visible soft-tissue contour defect, which is a major aesthetic concern, especially in women [1]. Restoration of facial volume and contour in this cosmetically sensitive region must be combined with the provision of a well-vascularized environment to support recovery of the preserved facial nerve. These challenges are further amplified when adjuvant radiotherapy is required, a frequent scenario in malignant parotid disease owing to close surgical margins along the facial nerve [2,3].

The principle of replacing “like with like” remains a central concept in reconstructive surgery. Various techniques have been described to restore parotid defects using muscle- and fat-based tissues [4,5,6]. Local flaps, including the superficial musculoaponeurotic system, temporoparietal fascia, temporalis muscle, and sternocleidomastoid muscle, are commonly employed but may fail to provide sufficient volume to adequately reconstruct both the superficial and deep components of a total parotidectomy defect [7,8]. Regional flaps, such as the supraclavicular and pectoralis major flaps, can provide additional tissue but often result in significant distortion of local anatomy, function, and aesthetics [9,10].

**Figure 1:**
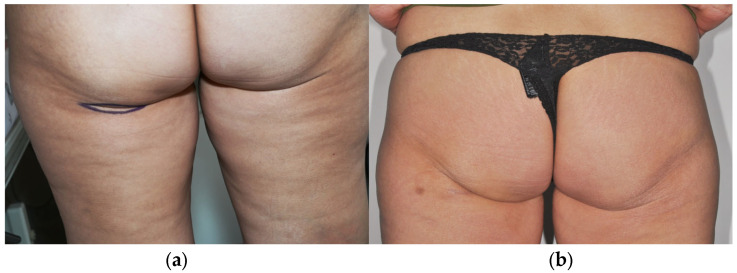
Inconspicuous infragluteal donor site for Adipofascial Infragluteal Perforator flap harvest. (**a**) Preoperative image. The lines indicate the skin incisions. (**b**) Six months postoperative aspect demonstrating concealment of the scar.

With an increasing incidence of malignant parotid tumors in younger patients, expectations regarding aesthetic outcomes have become more pronounced, particularly in beardless individuals. Consequently, patients often express high aesthetic demands for both the recipient and donor sites. Several free flaps, including the radial forearm free (RFF) flap, anterolateral thigh (ALT) flap, and scapular system flaps, as well as newer options such as the profunda artery perforator (PAP) flap, have been reported for parotid reconstruction [6,11,12]. While these techniques provide reliable tissue transfer, they are associated with conspicuous donor-site scars. In contrast, the infragluteal crease—used for harvesting the fasciocutaneous infragluteal (FCI) flap—is a highly concealed donor site, even when the patient is undressed (Figure 1).

The FCI flap is based on the terminal artery and vein of the infragluteal vessels [13]. The AIGP flap shares the same vascular basis but excludes cutaneous tissue.

The FCI flap has been primarily described for breast and perineal reconstruction [14,15,16]. Owing to its favorable tissue characteristics, including sufficient volume, appropriate consistency for facial contour restoration, reliable vascular anatomy, and a discreet donor site, this anatomical region may also be suitable for parotid reconstruction.

In this retrospective study, we describe the novel application of the AIGP free flap for reconstruction of total parotidectomy defects with skin and facial nerve preservation.

## 2. Patients and Methods

This report represents a preliminary consecutive case series designed to retrospectively evaluate the technical feasibility and early clinical outcomes of this reconstructive approach.

Three consecutive patients (one man and two women) with parotid gland tumors requiring total parotidectomy with preservation of the skin and facial nerve were treated between June and July 2025 at the Department of Oral and Maxillofacial Surgery, Medicover Hospital Cluj-Napoca, Romania and retrospectively analyzed. Preoperative diagnosis was established using contrast-enhanced magnetic resonance imaging and core-needle biopsy, revealing one case of recurrent pleomorphic adenoma, one low-grade mucoepidermoid carcinoma, and one high-grade mucoepidermoid carcinoma. In all cases, facial nerve function was intact preoperatively, and there was no tumor involvement of the overlying parotid skin. None of the patients had significant comorbidities, all were non-smokers, and none had a history of surgery in the buttock region. Patient age ranged from 29 to 56 years (mean, 43 years). The follow-up period was 7 months (Table 1).

### 2.1. Surgical Procedure

**Figure 2:**
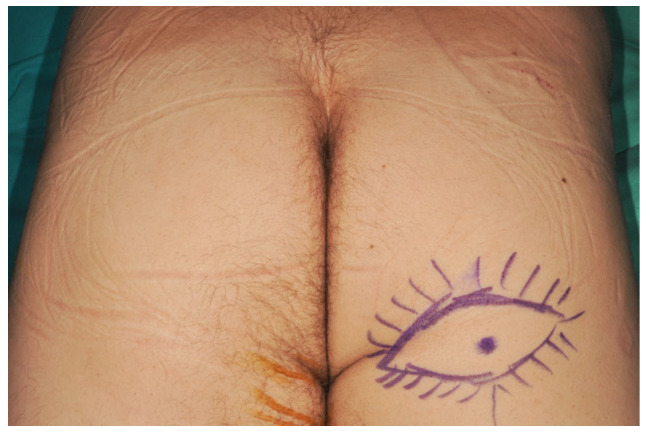
Marking points for AIGP flap harvest in the prone position. The continuous line represents the contour of the skin paddle incision. The radial lines represent the additional fat areas that will be harvested. The point indicates a cutaneous perforator.

Preoperative marking was performed with the patient in the prone position to identify cutaneous perforators arising from the descending branch of the inferior gluteal artery using an 8-MHz handheld Doppler probe. In most cases, the dominant perforator was located near the midline (Figure 2).

**Figure 3:**
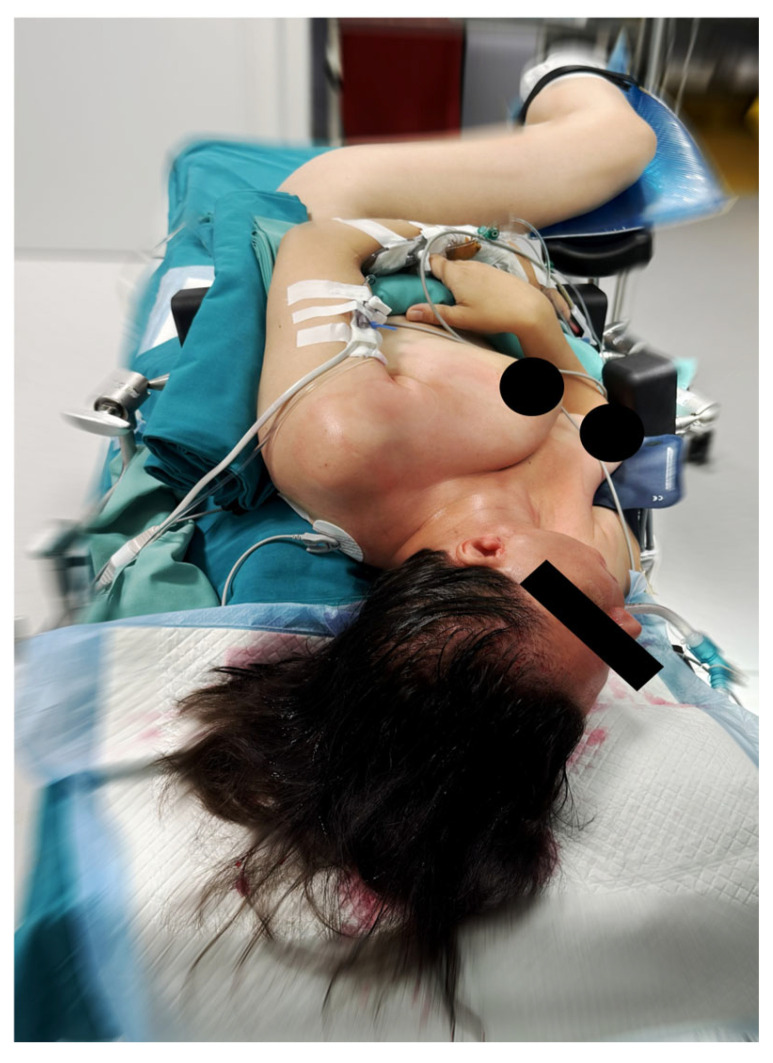
Position of the patient on the operating table that allows for a simultaneous two-team approach.

Surgery was performed under general anesthesia. The patient’s head was positioned laterally on the unaffected side, with the thorax rotated approximately 30° in the same direction and the pelvis placed in a lateral decubitus position. The ipsilateral buttock and posterior thigh served as the donor site for flap harvest. The lower limb was secured on a support with the knee flexed to approximately 60° (Figure 3).

This positioning enabled a simultaneous two-team approach, allowing concurrent parotidectomy and flap harvest.

**Figure 4:**
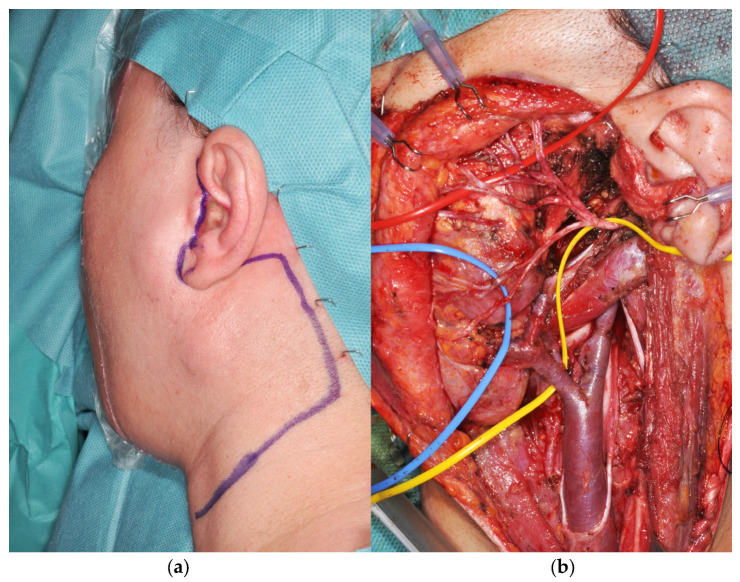
Intraoperative images of the patient with high-grade mucoepidermoid carcinoma: (**a**) Skin incision allowing tumor resection and neck dissection. (**b**) Preserved facial nerve: yellow loop—main trunk; red loop—temporo-facial division; blue loop—cervico-facial division.

Total parotidectomy was performed through a modified Blair incision, with the preauricular vertical limb positioned posterior to the tragus and extended into the mid-cervical crease when neck dissection was required (Figure 4a). In all cases, the overlying parotid skin and the functional facial nerve were preserved (Figure 4b).

Flap harvest was performed under 4× magnification loupes. The donor site was infiltrated subcutaneously with 10–15 mL of a lidocaine–epinephrine solution (1:200,000). A beveled incision was made approximately 2 cm above and below the infragluteal crease. Dissection was carried out obliquely using low-power electrocautery with a Colorado needle, progressing toward the gluteus maximus muscle and posterior thigh fascia to incorporate an adequate volume of adipofascial tissue. After identifying the muscle, dissection continued inferiorly to include the fascia within the flap. Intramuscular perforators from the gluteal artery were ligated. Perforators originating from the main pedicle were frequently associated with cutaneous nerve branches and were carefully preserved or divided as required. Dissection proceeded to the inferior border of the gluteus maximus muscle, after which the main perforators (typically two to three) were identified. The pedicle was then dissected proximally beneath the muscle toward the piriformis region, with careful protection of the posterior femoral cutaneous nerve. The sciatic nerve, located deep to the pedicle, was readily identified and protected. Adequate muscle relaxation facilitated retraction and pedicle exposure, and vascular clips were used to ligate collateral branches of the pedicle as needed.

**Figure 5:**
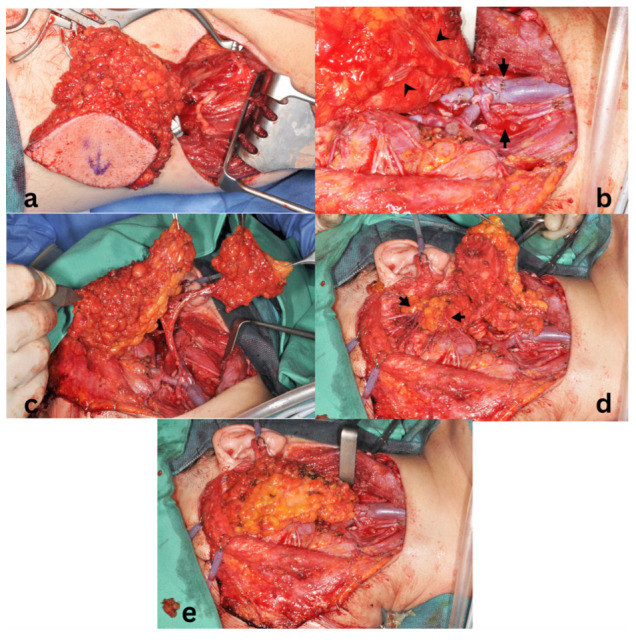
Adipofascial Infragluteal Perforator flap: (**a**) attached at the level of the buttock, with skin paddle still present. (**b**) Transferred and connected to the recipient cervical vessels: arrows—vascular anastomosis; arrowheads—terminal perforator vessels. (**c**) Tailored into two parts, each based on a perforator. (**d**) The smaller part of the flap placed under the facial nerve (arrows), to reconstruct the deep lobe of the parotid. (**e**) The larger part of the flap covering the facial nerve, restoring the anatomy of the parotid space.

After completion of the parotidectomy and flap dissection (Figure 5a), 3,000 IU of heparin was administered intravenously a few minutes prior to pedicle division. The flap was transferred to the neck, and microvascular anastomoses were performed to the selected recipient vessels (Table 1) using 7-0 and 8-0 polypropylene sutures (Prolene^®^, Ethicon, Johnson & Johnson, New Brunswick, NJ, USA) (Figure 5b). Following revascularization, the flap was tailored to the defect. The adipofascial tissue was divided into two components in a chimeric configuration, each based on an independent perforator, to reconstruct the superficial and deep parotid compartments (Figure 5c). The facial nerve was repositioned in its anatomical course, resting on the smaller, deep component of the flap and covered by the larger superficial component (Figure 5d,e).

**Figure 6:**
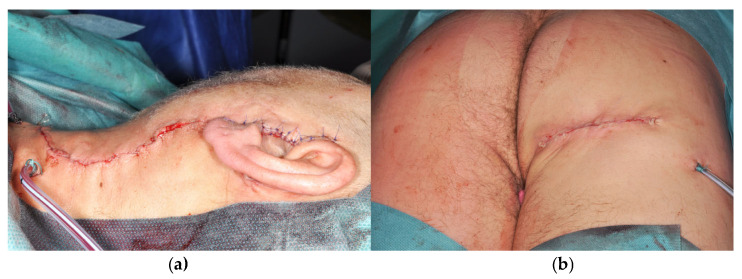
Final image after skin closure: (**a)** recipient site and (**b**) donor site.

The superficial portion of the flap was secured to the surrounding subcutaneous tissues with resorbable 4-0 polyglactin sutures (Vicryl^®^, Ethicon, Johnson & Johnson, New Brunswick, NJ, USA) and covered with the preserved skin. A closed-suction drain was placed in the subcutaneous plane of the neck and parotid region (Figure 6a). A skin marking was applied over the pedicle location to facilitate postoperative handheld Doppler monitoring.

The donor site was closed primarily in two layers over a closed-suction drain (Figure 6b).

Postoperatively, patients were positioned supine with the thorax elevated to 30° and the head maintained in a neutral position. Excessive forward flexion and lateral head rotation were restricted for two weeks. Prophylactic low-molecular-weight heparin and aspirin (75 mg daily) were administered during hospitalization, with aspirin continued for up to six weeks postoperatively. Cervical and donor-site drains were removed once output was less than 30 mL over 24 h.

Two patients with malignant tumors and positive surgical margins at the level of the facial nerve received adjuvant radiotherapy, which was initiated six weeks after surgery.

### 2.2. Patient Evaluation

The primary outcomes evaluated were flap survival—classified as complete survival, partial loss, or total failure—and the occurrence of major flap-related complications, including hematoma requiring surgical evacuation and postoperative infection. Secondary outcomes included facial nerve function, assessed using the House–Brackmann grading system at hospital discharge and at six months of follow-up; restoration of facial contour and symmetry at rest; and donor-site morbidity, including seroma formation, pain, functional limitations, and patient-reported perception of scar conspicuity.

Postoperative outcomes were assessed during routine follow-up visits through clinical examination performed by the surgical team. Aesthetic outcome and scar perception were also discussed with the patients during follow-up consultations, and overall satisfaction with the reconstructive result was recorded. Given the exploratory nature of this preliminary series and the limited number of cases, no validated aesthetic scoring systems or formal patient-reported outcome measures were applied.

Artificial intelligence-based tools used during the preparation of this manuscript did not analyze data, interpret the results, or influence clinical decision-making.

## 3. Results

**Figure 7:**
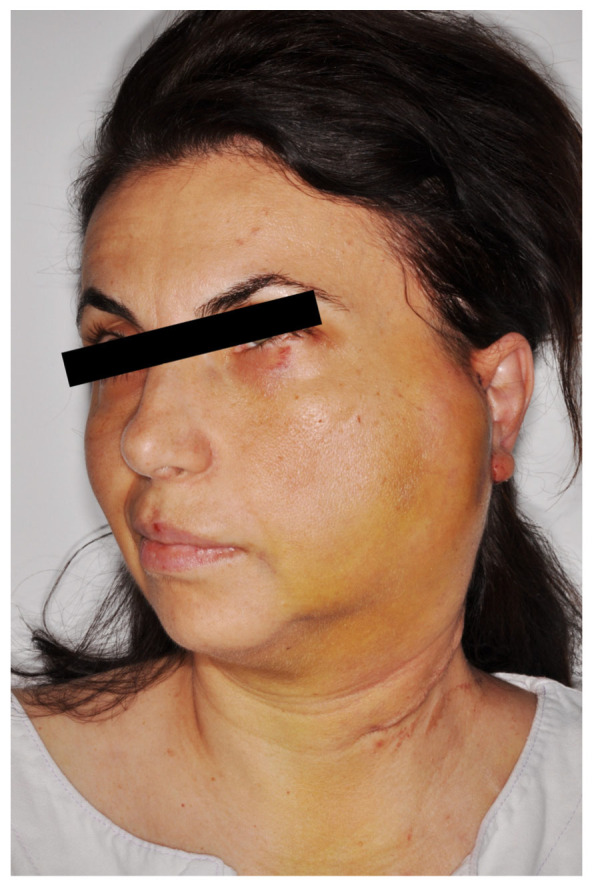
A patient with a diffuse hematoma at the reconstructed site (minor complication).

Successful reconstruction of total parotidectomy defects was achieved in all patients using an AIGP free flap. All three flaps demonstrated complete survival. No major flap-related complications occurred. One patient developed a postoperative hematoma involving the flap and parotid region, which was managed conservatively without the need for surgical intervention (Figure 7).

Minor donor-site seromas developed after drain removal in two patients and resolved with conservative management (Table 1). No wound dehiscence or surgical site infection was observed during hospitalization or follow-up.

Postoperative facial nerve function showed variable degrees of impairment immediately after surgery. Improvement in facial nerve function was observed in all patients by the six-month follow-up, including those who underwent adjuvant radiotherapy (Table 1). No clinically evident cases of Frey’s syndrome were identified during the available follow-up period.

**Figure 8:**
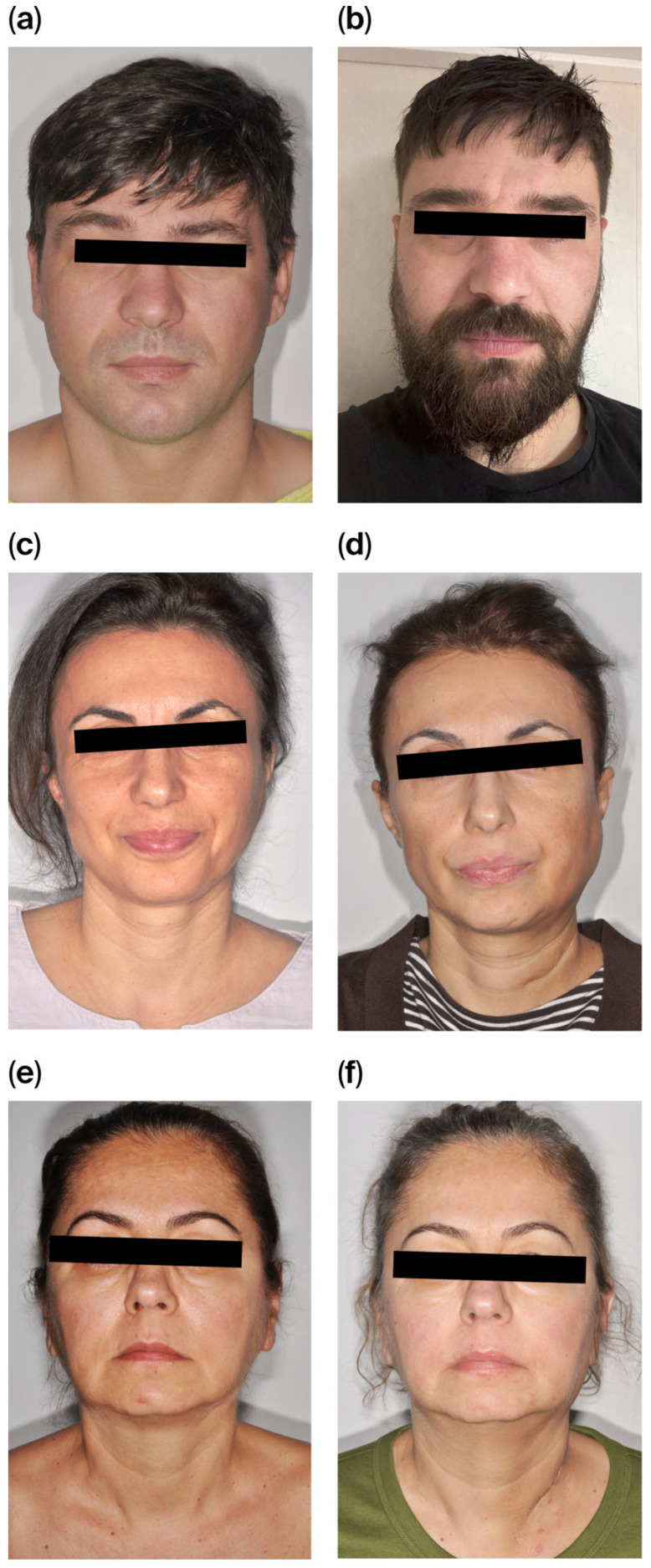
(**a**–**f**) Comparative aspects of the patients at six months follow up.

Adequate restoration of facial contour with protection of the preserved facial nerve was achieved in all cases. Mild volume overcorrection was noted in one patient. In patients who received postoperative radiotherapy, flap volume remained stable at six months, with minimal clinically apparent atrophy (Figure 8a–f). All patients reported satisfaction with their facial contour at the six-month follow-up.

Clinical examination during follow-up demonstrated satisfactory restoration of facial contour and symmetry at rest in all patients, with favorable scar appearance and high patient satisfaction regarding the aesthetic outcome. At the donor site, scars were perceived as inconspicuous by all patients at six months. No contour deformities, persistent pain, or functional limitations of the infragluteal region were reported.

These findings suggest that the technique is technically feasible and may represent a useful reconstructive option in selected cases.

## 4. Discussion

Total or extended parotidectomy commonly results in a visible depression of the lateral facial contour, often accentuating the ascending ramus and mandibular angle. Previous studies have reported high rates of cosmetic dissatisfaction among patients who do not undergo adequate volume and contour restoration following parotidectomy [17,18,19]. Consequently, reconstruction of total parotidectomy defects plays an important role in preserving patients’ self-image and social confidence, despite adding complexity to the surgical procedure.

Local flaps, including the superficial musculoaponeurotic system (SMAS) and sternocleidomastoid muscle (SCM) flaps, are frequently used but often fail to provide sufficient tissue volume to adequately reconstruct both the superficial and deep components of a total parotidectomy defect, and may be associated with functional compromise [20,21]. Regional flaps, such as the supraclavicular and pectoralis major flaps, can supply greater tissue volume but are commonly associated with donor-site deformities and scars that are unacceptable to many patients [22,23,24,25]. Free tissue transfer has therefore become a standard approach for complex parotid defects, offering a wide range of tissue characteristics and volumes [19,25,26]. In contemporary reconstructive practice, donor-site morbidity and scar conspicuity are increasingly important considerations, particularly in younger patients with active social and professional lives [27,28].

Perforator flaps represent a conceptual shift from traditional axial and regional flaps toward anatomy-preserving and function-sparing reconstructive solutions [29]. Among free flaps, the anterolateral thigh (ALT) flap remains a versatile workhorse for head and neck reconstruction [30], particularly when large volumes of soft tissue and skin are required. However, its bulk, long pedicle, and visible donor-site scar may limit its suitability for parotid reconstruction in selected patients. The profunda artery perforator (PAP) flap has emerged as an alternative, offering favorable contouring characteristics, but it is associated with a visible posterior thigh scar and may present variable vascular anatomy with relatively small-caliber vessels [12,31,32].

The AIGP flap shares its vascular pedicle with the fasciocutaneous infragluteal (FCI) flap, which has been widely used for pelvic, perineal, and breast reconstruction [13,14,16,33]. This donor region provides a substantial volume of adipofascial tissue with strong connective tissue support and a donor-site scar concealed within the infragluteal crease (Table 2). These characteristics suggest potential applicability for parotid reconstruction, where moderate volume replacement and soft-tissue pliability are required.

Although the use of the AIGP free flap for total parotidectomy reconstruction has not been previously reported, our experience indicates that this flap can provide pliable, well-vascularized adipofascial tissue without the bulk rigidity associated with muscle-based or thick fasciocutaneous flaps [13]. The ability to tailor the flap into a chimeric configuration allows separate reconstruction of the superficial and deep parotid compartments while accommodating the preserved facial nerve. By providing a vascularized tissue bed, the AIGP flap may offer a favorable environment for facial nerve recovery; however, this observation remains hypothesis generating and warrants further investigation.

In contrast to free fat grafting, which is associated with variable resorption and unpredictable long-term volume maintenance [34,35] vascularized adipofascial tissue may offer more stable volume over time. In our limited series, flap volume appeared clinically stable at six months, including in patients who underwent postoperative radiotherapy, although longer follow-up is required to assess late volume changes. As a muscle-sparing technique, the AIGP flap may also be associated with reduced donor-site morbidity.

Consistent with reports in breast reconstruction, substantial adipofascial tissue can be harvested from the infragluteal region without causing noticeable donor-site deformity [14,15]. The volume required for parotid reconstruction is considerably less than that needed for breast reconstruction, further supporting the suitability of this donor site. In our experience, donor-site scars were well concealed within the infragluteal crease, particularly in patients with buttock ptosis, and patient satisfaction with the donor site was high [36].

The ideal position for infragluteal flap harvest is the prone position with pelvic elevation [37]. In the present series, lateral positioning enabled a simultaneous two-team approach, allowing concurrent tumor resection and flap harvest. This approach increases technical difficulty during pedicle dissection, particularly beneath the gluteus maximus muscle where multiple collateral branches may be encountered, and therefore requires familiarity with the regional anatomy and microsurgical technique. The use of vascular clips may facilitate efficient and safe pedicle preparation in this setting.

Despite the limitations of a small sample size and short follow-up, our experience suggests that the AIGP free flap may be considered as an alternative option for reconstruction of total parotidectomy defects in carefully selected patients who prioritize an inconspicuous donor site, provided that an experienced microsurgical team is available. This series demonstrates the technical feasibility of combining parotid tumor resection and AIGP flap harvest within a simultaneous two-team approach.

Another limitation of the present study is the largely clinical and subjective assessment of aesthetic outcomes. No validated patient-reported outcome measures or standardized photographic evaluation systems were used. Future studies including larger patient cohorts should incorporate objective aesthetic evaluation tools and independent assessment in order to better characterize the reconstructive outcomes.

## 5. Conclusions

This initial clinical experience suggests that AIGP free flap appears to be a feasible and safe option for reconstruction of selected total parotidectomy defects. The flap provides well-vascularized adipofascial tissue with favorable characteristics for volume and contour restoration, allows on-site tailoring to the defect, and is associated with low donor-site morbidity and excellent scar concealment. In cases with facial nerve preservation, the AIGP flap may provide a supportive soft-tissue environment, even in the setting of adjuvant radiotherapy, while maintaining a muscle-sparing approach. The favorable early outcomes observed in this small preliminary series suggest potential reconstructive advantages, but larger studies with longer follow-up are required to further validate this approach.

## Figures and Tables

**Table 1:** Clinical characteristics and reconstructive outcomes of patients undergoing total parotidectomy reconstructed with adipofascial infragluteal perforator flap (AIGP).

	Age/Sex	Pathology	Surgery	Anastomosis/ Recipient Vessels	Complications	Adjuvant Radiation Therapy	Facial Nerve		Facial Contour/Flap Volume		Donor Site Scar Perception at Six Months Follow-Up
							Discharge	Six Months Follow-Up	Discharge	Six Months Follow-Up	
1	29/M	Recurrent pleomorphic adenoma	Total parotidectomy, facial nerve preservation	End-to-side/ECA End-to-side/TLF trunk	NO	NO	HB III	HB I	Adequate/Proper	Adequate/Proper	Inconspicuous
2	45/F	High grade muco-epidermoid carcinoma	Total parotidectomy, facial nerve preservation, ipsilateral selective neck dissection	End-to-end/FA End-to-side/IJV	Diffuse parotid hematoma Small gluteal seroma	YES	HB VI	HB II	Inadequate/Increased	Mild overcorrection/Acceptable	Inconspicuous
3	56/F	Low grade muco-epidermoid carcinoma	Total parotidectomy, facial nerve preservation, ipsilateral selective neck dissection	End-to-side/ECA End-to-side/IJV	Small gluteal seroma	YES	HB V	HB II	Adequate/Proper	Adequate/Proper	Inconspicuous

Abbreviations: ECA—External Carotid Artery; TLF—thyrolinguofacial trunk; FA—Facial Artery; IJV—Internal Jugular Vein; HB—House Brackmann scale.

**Table 2:** Fat type and characteristics depending on the area of harvest based on clinical observation and published literature.

Area	Fat Type	Firmness Level	Reason
Gluteal	Subcutaneous	Moderate	Thick layer with strong connective tissue anchoring.
Lower Belly	Subcutaneous	Low	Soft, mobile and pinchable with loose attachment.
Upper Belly	Visceral	High	Dense fat behind the abdominal wall; firm on palpation.
Inner Arms	Subcutaneous	Very Low	Thin, loose fat with minimal structural support.

## Data Availability

The data presented in this study are available on request from the corresponding author due to privacy and ethical restrictions.

## Abbreviations

The following abbreviations are used in this manuscript:

AIGP	Adipofascial Infragluteal Perforator
ALT	Anterolateral Thigh
ECA	External Carotid Artery
FA	Facial Artery
FCI	Fasciocutaneous Infragluteal
HB	House–Brackmann scale
IJV	Internal Jugular Vein
PAP	Profunda Artery Perforator
SCM	Sternocleidomastoid Muscle
SMAS	Superficial Musculoaponeurotic System
TLF	Thyrolinguofacial trunk

## References

[1] Wang S.J., Eisele D.W. (2012). Parotidectomy—Anatomical considerations. Clin. Anat..

[2] Guntinas-Lichius O., Silver C.E., Thielker J., Bernal-Sprekelsen M., Bradford C.R., De Bree R., Kowalski L.P., Olsen K.D., Quer M., Rinaldo A. (2018). Management of the facial nerve in parotid cancer: Preservation or resection and reconstruction. Eur. Arch. Otorhinolaryngol..

[3] Moore M.G., Yueh B., Lin D.T., Bradford C.R., Smith R.V., Khariwala S.S. (2021). Controversies in the Workup and Surgical Management of Parotid Neoplasms. Otolaryngol. Neck Surg..

[4] Thariat J., Carsuzaa F., Beddok A., Deneuve S., Marcy P.-Y., Merlotti A., Dejean C., Devauchelle B. (2024). Reconstructive flap surgery in head and neck cancer patients: An interdisciplinary view of the challenges encountered by radiation oncologists in postoperative radiotherapy. Front. Oncol..

[5] Chang B.A., Asarkar A.A., Horwich P.M., Nathan C.A.O., Hayden R.E. (2023). Regional pedicled flap salvage options for large head and neck defects: The old, the new, and the forgotten. Laryngoscope Investig. Otolaryngol..

[6] Elliott R.M., Weinstein G.S., Low D.W., Wu L.C. (2011). Reconstruction of Complex Total Parotidectomy Defects Using the Free Anterolateral Thigh Flap: A Classification System and Algorithm. Ann Plast Surg..

[7] Steuer C.E., Hanna G.J., Viswanathan K., Bates J.E., Kaka A.S., Schmitt N.C., Ho A.L., Saba N.F. (2023). The evolving landscape of salivary gland tumors. CA Cancer J. Clin..

[8] Irvine L.E., Larian B., Azizzadeh B. (2016). Locoregional Parotid Reconstruction. Otolaryngol. Clin. N. Am..

[9] Hanasono M.M., Matros E., Disa J.J. (2014). Important Aspects of Head and Neck Reconstruction. Plast. Reconstr. Surg..

[10] Bussu F., Gallus R., Navach V., Bruschini R., Tagliabue M., Almadori G., Paludetti G., Calabrese L. (2014). Contemporary role of pectoralis major regional flaps in head and neck surgery. Acta Otorhinolaryngol. Ital..

[11] Fatani B. (2023). Radial Forearm Free Flap for Head and Neck Defect Reconstruction: An Up-to-date Review of the Literature. Cureus.

[12] Elmorsi R., Lee Z.-H., Ismail T., Largo R.D. (2024). Profunda Artery Perforator Flaps in Head and Neck Reconstruction. Oral Maxillofac. Surg. Clin. N. Am..

[13] Scheufler O., Farhadi J., Kovach S.J., Kukies S., Pierer G., Levin L.S., Erdmann D. (2006). Anatomical Basis and Clinical Application of the Infragluteal Perforator Flap. Plast. Reconstr. Surg..

[14] Fodor L., Fodor M., Sobec R., Shiffman M.A. (2016). Fasciocutaneous Infragluteal Flap for Breast Reconstruction. Breast Reconstruction.

[15] Papp C., Windhofer C., Michlits W. (2011). Autologous Breast Augmentation With the Deepithelialized Fasciocutaneous Infragluteal Free Flap: A 10-Year Experience. Ann. Plast. Surg..

[16] Windhofer C., Michlits W., Gruber S., Papp C. (2010). Reconstruction in the buttock region using the local fasciocutaneous infragluteal (FCI) flap. J. Plast. Reconstr. Aesthet. Surg..

[17] Bianchi B., Ferri A., Ferrari S., Copelli C., Sesenna E. (2011). Improving Esthetic Results in Benign Parotid Surgery: Statistical Evaluation of Facelift Approach, Sternocleidomastoid Flap, and Superficial Musculoaponeurotic System Flap Application. J. Oral Maxillofac. Surg..

[18] Brady J.S., Lu G.N., Vila P.M., Rizvi Z.H. (2025). Patient and Surgeon-Reported Satisfaction With Reconstruction Following Superficial Parotidectomy: A Systematic Review and Meta-Analysis. Laryngoscope Investig. Otolaryngol..

[19] Moy J., Wax M.K., Loyo M. (2021). Soft Tissue Reconstruction of Parotidectomy Defect. Otolaryngol. Clin. N. Am..

[20] Vozel D., Pukl P., Groselj A., Anicin A., Strojan P., Battelino S. (2021). The importance of flaps in reconstruction of locoregionally advanced lateral skull-base cancer defects: A tertiary otorhinolaryngology referral centre experience. Radiol. Oncol..

[21] Ambro B.T., Goodstein L.A., Morales R.E., Taylor R.J. (2013). Evaluation of Superficial Musculoaponeurotic System Flap and Fat Graft Outcomes for Benign and Malignant Parotid Disease. Otolaryngol. Neck Surg..

[22] Chim H., Salgado C.J., Seselgyte R., Wei F.-C., Mardini S. (2010). Principles of Head and Neck Reconstruction: An Algorithm to Guide Flap Selection. Semin. Plast. Surg..

[23] Falade I.O., Murphy A.I., Switalla K.M., Yin R.R., Rose J.A. (2024). Functional donor-site morbidity following reconstruction with pectoralis major flaps: A systematic review. JPRAS Open.

[24] Hamidian Jahromi A., Horen S.R., Miller E.J., Konofaos P. (2022). A Comprehensive Review on the Supraclavicular Flap for Head and Neck Reconstruction. Ann. Plast. Surg..

[25] Louizakis A., Antoniou A., Kalaitsidou I., Tatsis D. (2025). Free Tissue Transfer Versus Locoregional Flaps for the Reconstruction of Small and Moderate Defects in the Head and Neck Region: A Narrative Review. Cureus.

[26] Galviz Tabares B., Ruiz Geithner C.M., Pierpoline J., Mosquera C. (2025). Long-Term Functional Outcomes of Free Flaps Versus Locoregional Flaps in Soft Tissue Reconstruction for Oral Cavity Cancer: A Systematic Review. J. Craniofac. Surg..

[27] McLeod G.J., Islur A. (2020). Donor Site Scar Preference in Patients Requiring Free Flap Reconstruction. Plast. Surg..

[28] Russell J., Pateman K., Batstone M. (2021). Donor site morbidity of composite free flaps in head and neck surgery: A systematic review of the prospective literature. Int. J. Oral Maxillofac. Surg..

[29] Dandoulakis E. (2025). Advances in perforator flap use for head and neck reconstruction: A systematic review of clinical outcomes and innovations. World J. Biol. Pharm. Health Sci..

[30] Pu J.J., Atia A., Yu P., Su Y.X. (2024). The Anterolateral Thigh Flap in Head and Neck Reconstruction. Oral Maxillofac. Surg. Clin. N. Am..

[31] Marchi F., Iandelli A., Pace G.M., Bellini E., Tirrito A., Costantino A., Cerri L., Greco A., Polimeni A., Parrinello G. (2025). Surgical outcomes of profunda artery perforator flap in head and neck reconstruction: A systematic review and meta-analysis. Head Neck.

[32] Wilson R., Cave T., Entezami P., Ware E., Chang B.A. (2024). Systematic Review of the Profunda Artery Perforator Free Flap for Head and Neck Reconstruction. OTO Open.

[33] Michlits W., Windhofer C., Papp C. (2009). Pectus Excavatum and Free Fasciocutaneous Infragluteal Flap: A New Technique for the Correction of Congenital Asymptomatic Chest Wall Deformities in Adults. Plast. Reconstr. Surg..

[34] Tamplen M., Knott P.D., Fritz M.A., Seth R. (2016). Controversies in Parotid Defect Reconstruction. Facial Plast. Surg. Clin. N. Am..

[35] Fawzy A., Balbaa M.A., Hagag M. (2023). Evaluation of functional and aesthetic outcomes of free dermal fat graestheticuperficial musculoaponeurotic system flap after superficial parotidectomy: Randomized clinical trial. BJS Open.

[36] Mughal M., Baker R., Muneer A., Mosahebi A. (2013). Reconstruction of perineal defects. Ann. R Coll. Surg. Engl..

[37] Papp C., Michlits W., Gruber S. (2007). The fasciocutaneous infragluteal free flap (FCI): A new approach in breast reconstruction. Eur. Surg..

